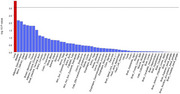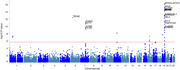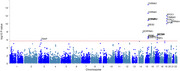# Common Genetic Variants and Pathways Between COPD and AD: Insights from a GWAS of Over 300,000 Individuals in UK Biobank

**DOI:** 10.1002/alz70862_109928

**Published:** 2025-12-23

**Authors:** YiMeng Ren, Qin Chen

**Affiliations:** ^1^ west china hospital, chengdu, sichuan China; ^2^ West China Hospital of Sichuan University, Chengdu, Sichuan China

## Abstract

**Background:**

The etiology of Alzheimer’s disease (AD) is intricate pathological mechanism. Notably, extensive researches has demonstrated the significant involvement of chronic hypoxia in AD progression.

**Method:**

To identify potential gentic association of outcome AD with exposure index lung functions, Mendelian randomization (MR)analysis was performed. In this study, data from Catalog GWAS involving over 25,392 patients with AD was regarded as outcome and data from IEU containing 1,887,658 participants with lung function tests was used as exposures. An additional genome‐wide association studys (GWAS) using the Affymetrix UK BiLEVE Axiom array was performed to validate the risk SNPs (pval<0.5) in UK Biobank phenotypic data and WES genetic data, involving 57795 case of AD, 69884 cases of COPD and over 270,000 controls. The novel machine‐learning app “REGENIE” with whole‐genome regression model for quantitative and binary phenotypes was performed for the association testing. Extensive quality‐control methods were performed at both the sample and the SNP level. To further explore the functional implications of the identified SNPs, we utilized FUMA GWAS, a web‐based platform designed for functional mapping and annotation of genome‐wide association study results.

**Result:**

MR results showed lung function like forced vital capacity (FVC) (OR=0.83, 95%CI: 0.77‐0.92 and forced expiratory volume in 1‐second (FEV1) (OR=0.86, 95%CI: 0.77‐0.96) may be protective factors for AD. Further investigation of GWAS counted 49 significant risk SNPs in AD (*p* <1x10‐8) and 25 risk SNPs s in COPD (*p* <1x10‐8). Four highly associated signals of overlapping risk loci (TOMM40_rs11556505, AOPE_ rs429358, APOE_rs769449, TOMM40_rs157581) with p value < 1x10‐8, and other three SNPS (TOMM40_rs184017, TOMM40_rs2075650, TOMM40_rs157582) with p value< 5x10‐8 for both AD and COPD were found. MAGMA gene‐property analysis revealed significant enrichment of Alzheimer’s disease‐associated SNPs in gene expression specific to lung tissue (*p* < 10^−3^), highlighting the potential involvement of respiratory system pathways in disease etiology.

**Conclusion:**

The seven identified risk loci highlight the shared roles of potential pathogenic gene polymorphisms between COPD and AD. These novel candidate genes provide further evidence supporting the overlapping genetic susceptibility between the two diseases. Additionally, FUMA GWAS analysis of AD revealed a strong genetic correlation with COPD, with seven significant SNPs shared across the GWAS results of both conditions.